# Quantitative T2*-Weighted Imaging and Reduced Field-of-View Diffusion-Weighted Imaging of Rectal Cancer: Correlation of R2* and Apparent Diffusion Coefficient With Histopathological Prognostic Factors

**DOI:** 10.3389/fonc.2021.670156

**Published:** 2021-05-24

**Authors:** Yang Peng, Yan Luo, Xuemei Hu, Yaqi Shen, Daoyu Hu, Zhen Li, Ihab Kamel

**Affiliations:** ^1^ Department of Radiology, Tongji Hospital, Tongji Medical College, Huazhong University of Science and Technology, Wuhan, China; ^2^ Tongji Hospital, Tongji Medical College, Huazhong University of Science and Technology, Wuhan, China; ^3^ Department of Radiology and Radiological Sciences, Johns Hopkins University, Baltimore, MD, United States

**Keywords:** rectal neoplasms, diffusion weighted imaging, magnetic resonance imaging, prognosis, biomarkers

## Abstract

**Purpose:**

To assess T2*-weighted imaging (T2*WI) and reduced field-of-view diffusion-weighted Imaging (rDWI) derived parameters and their relationships with histopathological factors in patients with rectal cancer.

**Methods:**

Fifty-four patients with pathologically-proven rectal cancer underwent preoperative T2*-weighted imaging and rDWI in this retrospective study. R2* values from T2*-weighted imaging and apparent diffusion coefficient (ADC) values from rDWI were compared in terms of different histopathological prognostic factors using student’s t-test or Mann-Whitney U-test. The correlations of R2* and ADC with prognostic factors were assessed by Spearman correlation analysis. The diagnostic performances of R2* and ADC were analyzed by receiver operating characteristic curves (ROC) separately and jointly.

**Results:**

Significant positive correlation was found between R2* values and T stage, lymph node involvement, histological grades, CEA level, the presence of EMVI and tumor deposit (r = 0.374 ~ 0.673, p = 0.000–0.006), with the exception of CA19-9 level, CRM status and tumor involvement in the circumference lumen (TIL). Meanwhile, ADC values negatively correlated with almost all the prognostic factors (r = −0.588 to −0.299, p = 0.000–0.030), except CA19-9 level. The AUC range was 0.724–0.907 for R2* and 0.674–0.887 for ADC in discrimination of different prognostic factors. While showing the highest AUC of 0.913 (0.803–1.000) in differentiation of T stage, combination of R2* and ADC with reference to different prognostic factors did not significantly improve the diagnostic performance in comparison with individual R2*/ADC parameter.

**Conclusions:**

R2* and ADC were associated with important histopathological prognostic factors of rectal cancer. R2* might act as additional quantitative imaging marker for tumor characterization of rectal cancer.

## Introduction

Colorectal cancer (CRC) is presently the most common tumor occurring in the digestive system, with high mortality worldwide (1). Approximately 30% to 35% of CRCs arise from the rectum (2). The prognosis of rectal cancer is associated with many factors, such as TNM staging, histological differentiation, extramural vascular invasion (EMVI), circumferential margin (CRM) involvement, range of tumor involvement in the circumference lumen (TIL) and tumor markers including carcinoembryonic antigen (CEA) and cancer antigen 19-9 (CA19-9) (3–7). The choice of treatment including surgery, with or without preoperative and postoperative neoadjuvant chemo-radiotherapy depends on the probability of patients having distant metastasis and local recurrence (8–10). Thus, the risk stratification for distant metastasis and tumor recurrence based on the prognostic factors is very important for treatment planning of patients with rectal cancer.

Current MRI techniques of rectal cancer utilized in clinics are comprised of T2 weighted imaging, diffusion-weighted imaging (DWI), dynamic-contrast-enhanced imaging technique (DCE), and other functional techniques. For instance, as for MR functional technique, some report (11) indicated that IVIM parameters, f, D and D* can reflect clinically relevant histopathological features in rectal cancer. However, some MRI techniques are inherent with inadequacies. The administration of contrast media is required for DCE-MRI by invasive method. As for DWI technique, it is based on single-shot echo-planar imaging (ss-EPI), which is often associated with limited image quality including geometric distortion, ghosting and insufficient image resolution (12, 13). Therefore, optimization of MRI techniques is necessitated for rectal cancer imaging.

Reduced field-of-view (FOV) diffusion-weighted imaging (rDWI), utilizes a 2D spatially selective echo-planar radiofrequency pulse, which is followed by 180°refocusing pulse (14). The rDWI technique decreases the off-resonance- related artifacts and blurring by reducing FOV in the phase-encoding direction of EPI readout to decrease the number of k-space lines. The rDWI technique has been studied to improve overall image quality and diagnostic performance, as compared to conventional DWI using ss-EPI (15). Previous studies reported successful application of rDWI technique for imaging of breast cancer, prostate cancer and endometrial cancer (16–18).

On the other hand, T2*-weighted imaging (T2*WI), a multi-echo gradient-echo sequence, requires a magnetic field with high uniformity, which is helpful for improvement of detection of small lesions with comparatively high resolution (19). Besides, the time needed for T2*WI scanning is short and it is very convenient to perform analysis of images on the post-processing workstation. T2*WI is closely associated with the oxygenation status of hemoglobin, which could affect the homogeneity of magnetic field, resulting in signal transformation on T2*WI (20). The apparent relaxation rate R2* from T2*WI (R2* = 1/T2*), is related to the partial pressure of oxygen and deoxyhemoglobin content. Elevated levels of deoxyhemoglobin causes elevated R2* value (21). R2* value was utilized in previous studies (22–24) to noninvasively evaluate tumor biological behaviors in renal cancer, breast cancer, and cervical cancer.

Therefore, we hypothesize R2* parameter might be correlated with histopathological prognostic factors of rectal cancer. To our knowledge, the relationship between tumor R2* and histopathological prognostic factors has not been reported. Thus, the purpose of our study was to assess the potential role of R2* from T2*-weighted imaging and ADC from rDWI in histopathological prognosis of patients with rectal cancer undergoing primary surgery.

## Materials and Methods

### Patients

**Figure 1:**
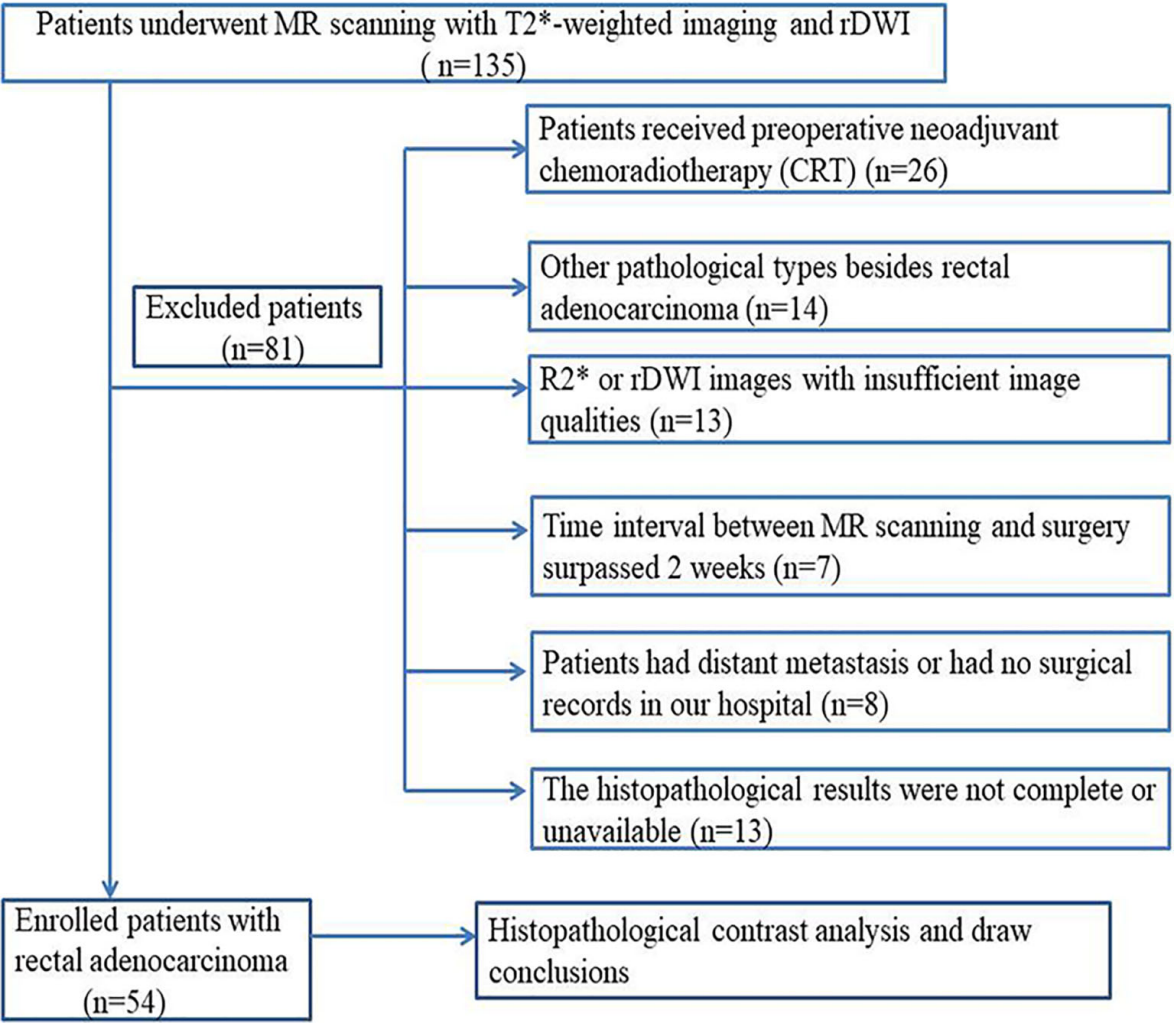
Flowchart of patient selection process by exclusion criteria.

This retrospective study was approved by our Institutional Review Board and the requirement for written informed consent of patients with rectal cancer was waived. Between September 2016 and November 2017, a series of 135 consecutive patients with rectal cancer verified by endoscopic biopsy were referred for MR examinations for individual treatment planning. The inclusion and exclusion criteria are shown in **Figure 1**. Finally, 54 patients (39 men, 15 women; aged 57 ± 11 years; range 39–82 years) were enrolled in this retrospective study. The median time interval between MR imaging and surgery was 4 days (range, 2–6 days).

### MRI Imaging Protocol

An intramuscular injection of 5 mg racanisodamine hydrochloride was administered to reduce intestinal peristalsis 30 min before MR examination. All patients underwent MR scanning of the whole pelvis in the supine position with a 3.0T MR unit (Discovery 750; GE Healthcare, Milwaukee, WI), which was equipped with 32-channel phased-array torso coil. The standardized MR protocol for imaging rectal cancer was comprised of axial T1-weighted fast spin echo (FSE) and T2-weighted FSE sequences without fat saturation in three directions. Besides, T2*-weighted imaging and rDWI (b values, 0 and 800 s/mm^2^) sequences were also included in the imaging protocol. The protocol details are listed in **Table 1**.

**Table 1:** MR scanning protocol parameters.

Parameters	T1WI	T2WI	rDWI	T2*WI
Sequence	FSE	FSE	EPI	Multiecho GRE
Orientation	Oblique axial	Oblique axial, sagittal and coronal	Oblique axial	Oblique axial
Repetition time (msec)	500	5629	4000	100
Echo time (msec)	75	85	75	2.7, 6.8, 10.9, 15.1, 19.2, 23.3, 27.4, 31.5, 35.6, 39.7, 43.8, 48.0, 52.1
FOV (mm^2^)	380×380	200×200	200×100	240×192
Matrix (mm^2^)	320×224	448×314	128×64	192×160
Slice thickness (mm)	5	3	3	3
Slice gap (mm)	1	0	0	0
b-value (s/mm^2^)	N/A	N/A	0, 800	N/A
Bandwidth (kHz)	62.50	31.3	250	31.3
No. of signals acquired	2	4	12	1

GRE, gradient-recalled echo; EPI, echo planar imaging; FSE, fast spin-echo; FOV, field-of-view.

### Imaging Analysis

All the MR images including T2*-weighted imaging and rDWI were uploaded to an Advantage Workstation (version 4.6, GE healthcare, USA). The ADC values from rDWI data were measured on ADC maps with the Functool-ADC software. Post-processing of R2* values was performed with the Functool-R2Star software (GE healthcare, USA). Two independent radiologists with 7 and 15 years of experience in MRI diagnosis of gastrointestinal diseases interpreted all the images independently, and they were blinded to all clinical and pathological information.

The R2* value from T2*-weighted imaging was obtained from the Functool-R2Star software. The distribution of R2* was displayed in an axial R2* color map. The R2* and T2* maps for each lesion of rectal cancer were measured automatically by fitting a single exponential model of the ln (signal intensity) to TE curve. The R2* (R2*=1/T2*) value was determined by the slope of the ln (signal intensity) versus TE by using 13 different data points (TE = 2.7, 6.8, 10.9, 15.1, 19.2, 23.3, 27.4, 31.5, 35.6, 39.7, 43.8, 48.0, 52.1 ms) for T2*-weighted imaging. The region-of-interests (ROIs) were manually drawn along the border of the rectal tumor on each consecutive R2* and ADC maps covering the whole lesion. All ROIs were placed on the solid region of tumor to avoid cystic, necrotic and hemorrhagic areas. ROI delineations were determined utilizing T2 weighted images and diffusion weighted images as references for R2* and ADC measurements. Tumor volume, R2* and ADC measurements were performed by two different abdominal radiologists and were compared to evaluate inter-reader agreement.

### Histopathologic Assessment

The histopathological diagnosis of resected surgical specimens was performed by a gastrointestinal pathologist with over 20 years’ experience, and he was blinded to clinical information and MRI imaging data. The resected specimens were stained by hematoxylin and eosin for histopathological assessment. The final histopathological reports were comprised of the T category (the depth of tumor invasion), N category (lymph node metastasis), histological differentiation (well, moderately and poorly differentiated), CRM involvement, EMVI, TIL involvement and tumor deposits. The T/N categories of rectal cancer were evaluated and determined by the 7th edition of TNM staging system recommended by the American Joint Committee on cancer (AJCC) (25). Besides, the status of CRM involvement, EMVI and tumor deposits was recorded as present or absent. The assessment of TIL involvement was based on the invasion of bowel circumference (**Table 2**).

**Table 2:** Comparisons of R2* and apparent diffusion coefficient between different histopathological categories of rectal cancer.

Histopathological factor	N (%)	R2* (Hz)	p-value	ADC (×10^−3^ mm^2^)	p-value
pT category^▲^					
pT1+2	19 (35.2)	31.05 ± 9.58	**<0.001**	1.05 ± 0.26	**<0.001**
pT3+4	35 (64.8)	47.10 ± 7.76		0.91 ± 0.06	
pN category^☆^					
pN0	30 (55.6)	37.86 ± 11.58	**0.005**	0.99 ± 0.21	**0.030**
pN1+2	24 (44.4)	45.94 ± 9.59		0.92 ± 0.09	
Tumor grade^§^					
G1+2	39 (72.2)	38.23 ± 9.50	**0.001**	0.99 ± 0.19	**<0.001**
G3	15 (27.8)	49.83 ± 11.91		0.88 ± 0.07	
CEA level					
< 5mg	36 (66.7)	38.08 ± 10.69	**0.005**	0.99 ± 0.20	**0.001**
≥ 5mg	18 (33.3)	48.20 ± 9.82		0.88 ± 0.05	
CA19-9 level					
< 34 U/ml	43 (79.6)	40.09 ± 11.17	0.080	0.97 ± 0.19	0.125
≥ 34 U/ml	11 (20.4)	46.76 ± 11.16		0.90 ± 0.07	
CRM					
Negative	47 (87.0)	40.29 ± 11.02	0.062	0.97 ± 0.18	**0.004**
Positive	7 (13.0)	49.23 ± 11.54		0.86 ± 0.05	
EMVI					
Negative	41 (75.9)	38.31 ± 9.72	**<0.001**	0.99 ± 0.18	**<0.001**
Positive	13 (24.1)	51.37 ± 10.85		0.87 ± 0.07	
TIL^★^					
C1+2	13 (24.1)	35.96 ± 10.23	0.053	1.08 ± 0.15	**0.004**
C3+4	41(75.9)	43.19 ± 11.29		0.92 ± 0.16	
Tumor deposit					
Negative	42 (77.8)	38.83 ± 10.02	**0.006**	0.98 ± 0.18	**<0.001**
Positive	12 (22.2)	50.64 ± 11.49		0.86 ± 0.06	

^▲^1 patient was T1 stage, 18 patients were T2 stage, 28 patients were T3 stage and 7 patients were T4 stage.

^☆^12 patients had N1 stage and N2 stage, respectively.

^§^G1+2 indicated well/moderately-differentiated rectal cancer, and G3 indicated poor-differentiated rectal cancer (the number for G1 and G2 was 4 and 35, respectively).

^★^C1 indicated the tumor invasion was within 1/4 of the involved bowel circumference (the number for C1 was 0); C2 indicated the tumor invasion was >1/4 and ≤1/2 of the involved bowel circumference (the number for C2 was 13); C3 indicated the tumor invasion was >1/2 and ≤3/4 of the involved bowel circumference (the number for C3 was 9); C4 indicated the tumor invasion was >3/4 of the involved bowel circumference (the number for C4 was 32).

CRM , circumferential margin; EMVI, extramural vascular invasion; TIL, range of tumor involvement in the circumference lumen; ADC, apparent diffusion coefficient; N(%), the percentage of patients.

The bolded numbers in the “p-value” columns indicated there were significant differences.

### Clinical Tumor Markers

Clinical tumor markers were reviewed from the medical records of patients with rectal cancer. The plasma levels of CEA and CA199 were tested within 4 to 7 days before surgery. A CEA level greater than 5 ng/ml or a CA199 level greater than 34 U/ml in our hospital was considered elevated.

### Statistical Analysis

All statistical analyses were performed by utility of SPSS 19.0 (IBM, Armonk, NY, USA) and MedCalc software (version 12.7.0.0, Mariakerke, Belgium). The inter-observer variability of R2* and ADC parameters between the two radiologists was assessed by intraclass correlation coefficients (ICCs, range and correlation: 0.00–0.20, poor; 0.21–0.40, fair; 0.41–0.60, moderate; 0.61–0.80, good; 0.81–1.00, excellent) and the Bland-Altman analysis.

The Kolmogorov-Smirnov test and Levene test were utilized to assess whether the data from each histopathological group were in normal distribution or not. The Mann-Whitney U-test or independent-samples t-test was performed to assess differences between the following pairwise groups: pT1-2 versus pT3-4, pN0 versus pN1-2, well versus moderate/poor differentiation, normal level of CEA versus elevated level of CEA, normal level of CA199 versus elevated level of CA199, the presence versus absence of CRM, the presence versus absence of EMVI, TIL involvement (C1+C2 versus C3+C4) and the presence versus absence of tumor deposits. Nonparametric ANOVA analysis was utilized to compare tumor R2* and ADC values between different TIL levels. Adjustments were made by Bonferroni corrections for multiple testing between R2*/ADC parameters in association with different prognostic factors of rectal cancer. The Spearman correlation analysis was performed to assess the relationships between the prognostic factors and MRI parameters of R2* and ADC from patients with rectal cancer.

Receiver operating characteristic (ROC) curves were used to evaluate the diagnostic performances of R2* and ADC parameters in relation to various status of prognostic factors. The optimal cutoff value was selected regarding the Youden index. The areas under the curves (AUCs) were compared to analyze different diagnostic performances of R2* and ADC parameters. The two MRI parameters (R2* and ADC) were also combined using the binary regression analysis for ROC analysis. Pairwise comparison of ROC curves between individual R2*/ADC and combination of both parameters were performed by Z statistics. p<0.05 was considered to have statistical significance.

## Results

### Histopathological Findings

The patient characteristics and associated histopathological findings are shown in **Table 2**. In our study, 47 patients (T1-T3 stage) received radical surgery of rectal cancer (14 patients underwent Miles operation and 33 patients had Dixon surgery). The remaining 7 patients (T4 stage) underwent palliative surgery including tumor resections before receiving chemo-radiotherapy because of emergent complications (3 patients had intestinal obstruction, 2 patients had massive intestinal bleeding and 2 patients had persistent perianal pain, which could not be relieved by painkillers). Mean whole-tumor volume of rectal cancer is 9.76 ± 6.25 cm^3^.

### Interobserver Agreement

**Figure 2:**
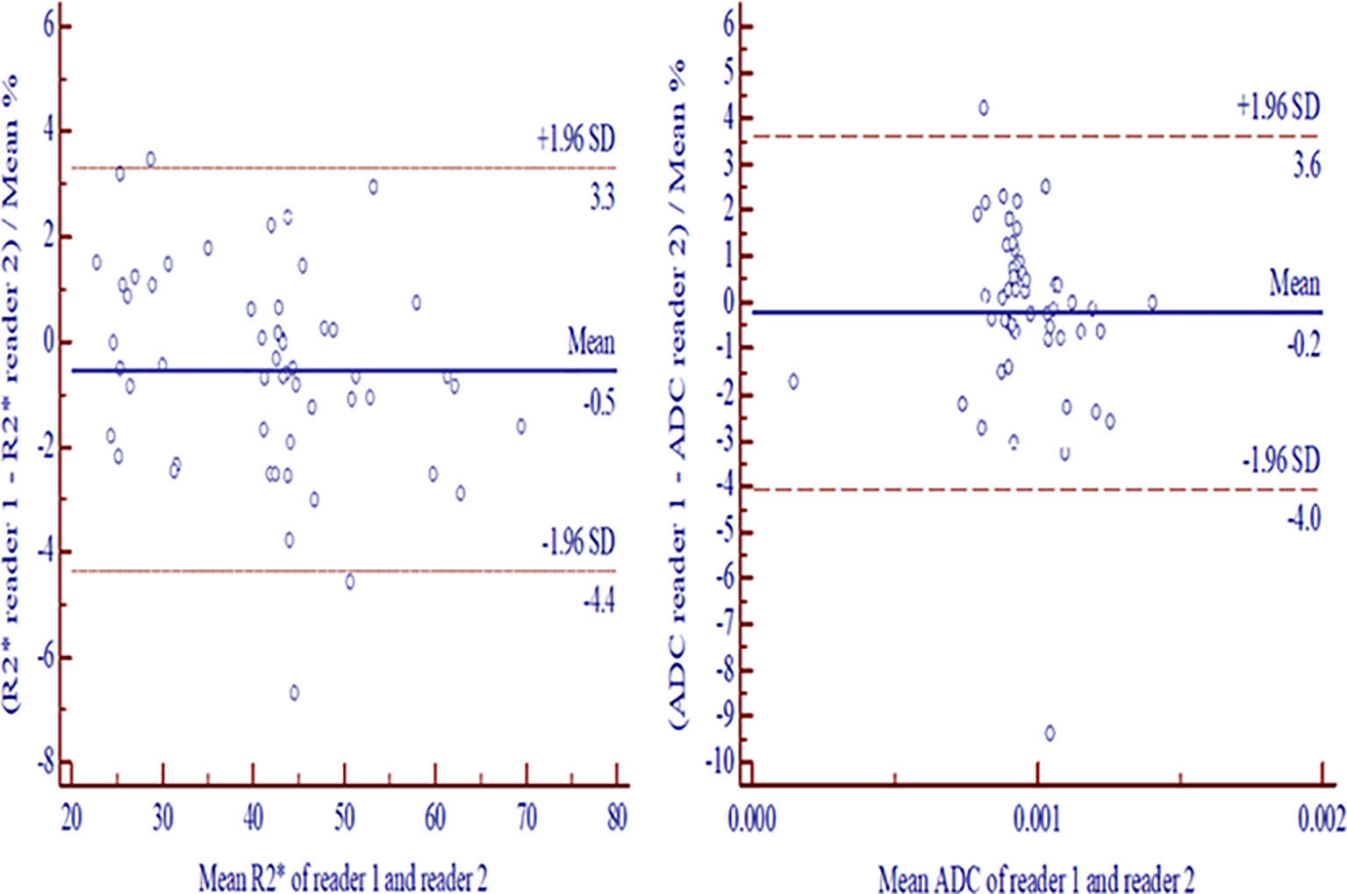
Bland-Altman plots exhibited interreader consistencies with 95% confidence intervals for measurement differences between two readers in R2* **(A)** and ADC **(B)**. ADC, apparent diffusion coefficient.

The interobserver agreements between readers were excellent for tumor volume (0.998, 95% CI: 0.996–0.999), measurements of R2* (0.999, 95%CI: 0.998–0.999) and ADC (0.997, 95%CI: 0.995–0.998). No significant differences were found for tumor volume between readers (p= 0.973). The Bland-Altman plots indicated the 95% limits of interobserver consistency were −4.4% to 3.3% (R2*) and −4.0% to 3.6% (ADC), respectively (**Figure 2**).

### Correlation of R2* and ADC Parameters With Histopathological Prognostic Factors

**Figure 3:**
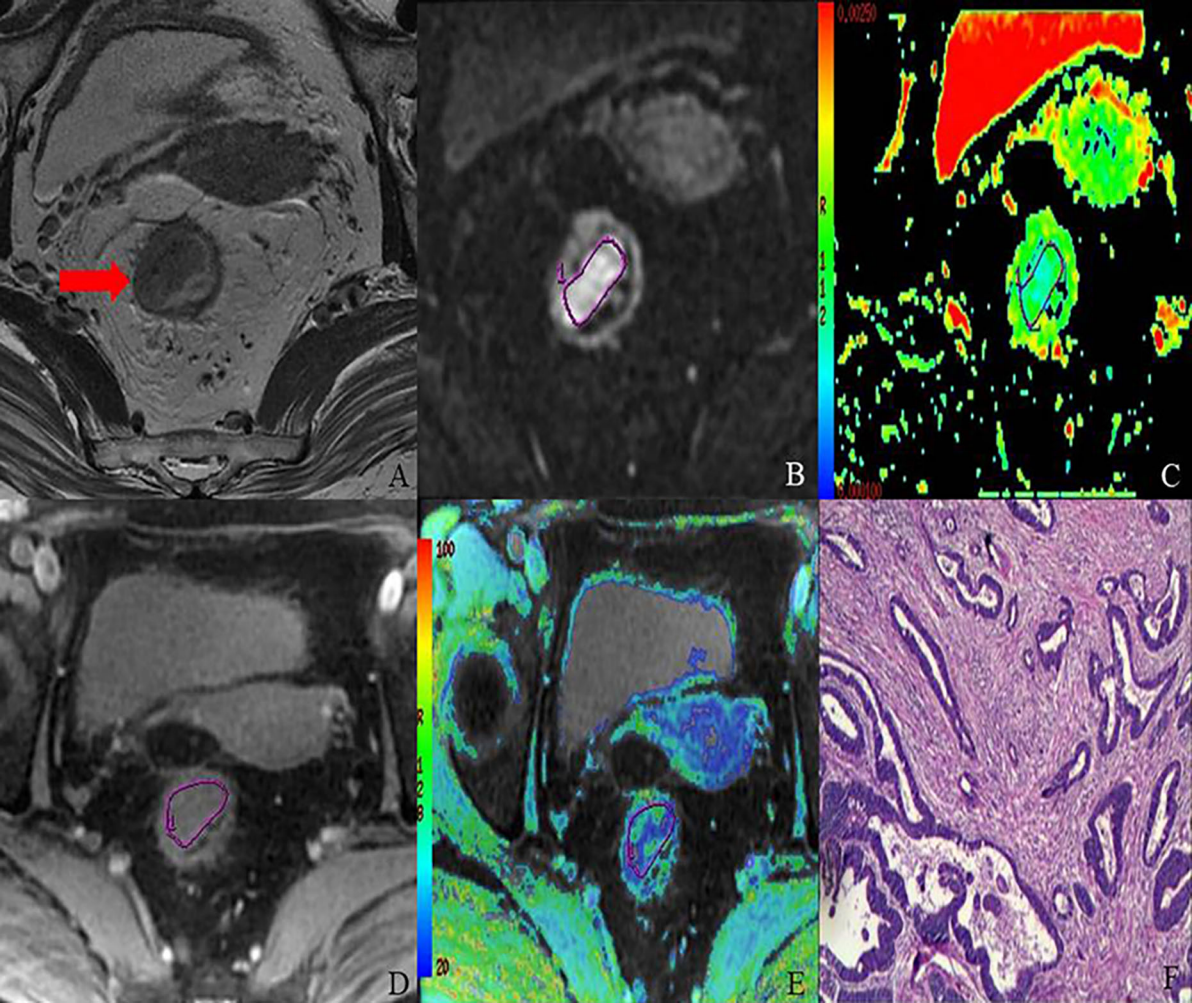
MR images of 68-year-old female with T2 stage rectal adenocarcinoma (well-differentiated; N1; location, middle). Axial T2-weighted imaging **(A)** shows intermediate-signal-intensity mass occupying more than one-half of the rectal wall (TIL, C3). rDWI image (b = 800 s/mm^2^) **(B)**, ADC color map (ADC= 1.05×10^−3^ mm^2^) **(C)**, T2*-weighted image **(D)** and R2* color map (R2*= 33.18 Hz) **(E)** show ROI delineations of the corresponding tumor. The histopathological specimen **(F)** shows well-differentiated rectal adenocarcinoma invading deep muscular layer of rectal wall.

**Figure 4:**
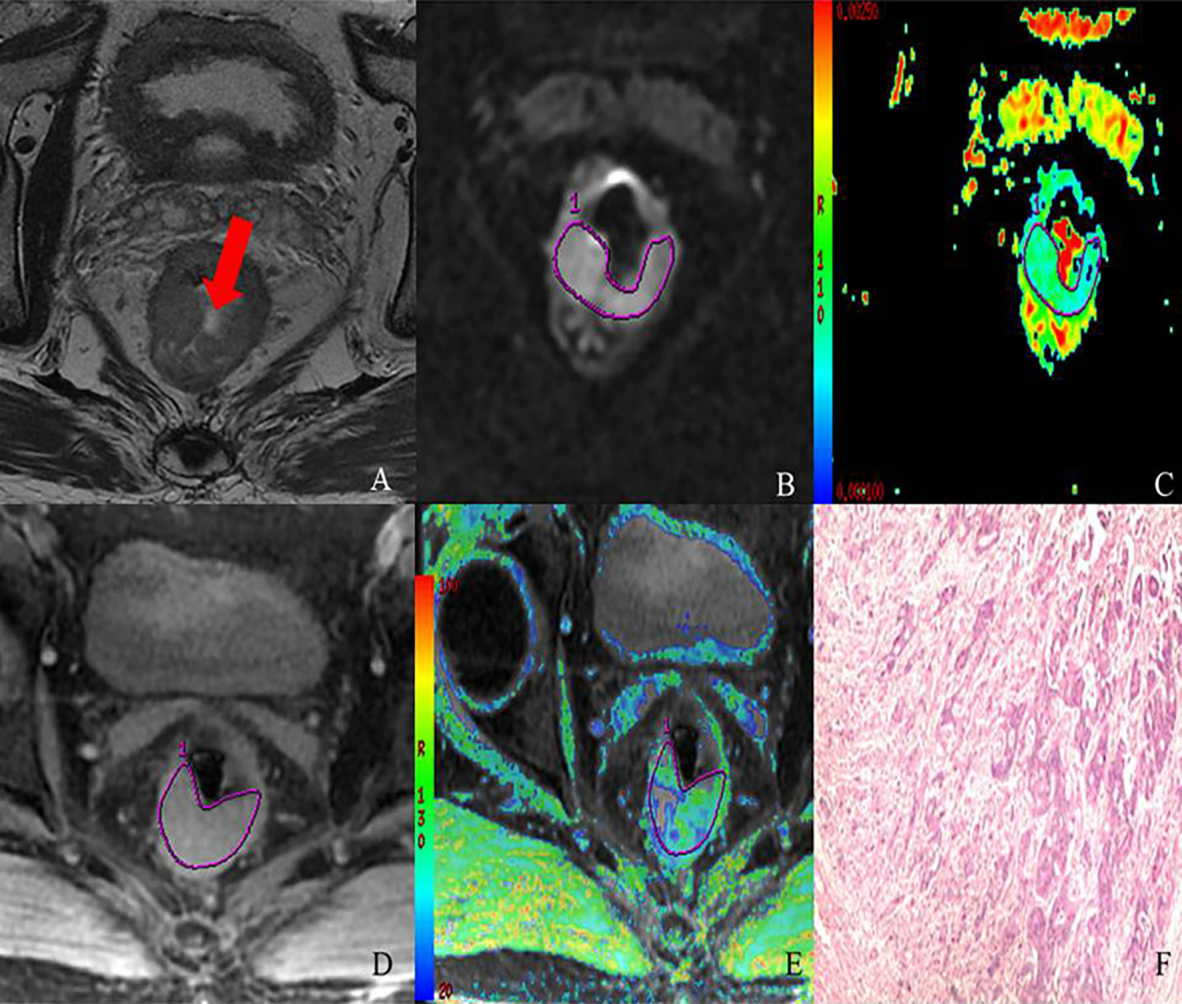
MR images of 69-year-old male with T3 stage rectal adenocarcinoma (moderately-differentiated; N1; location: middle). Axial T2-weighted imaging **(A)** shows intermediate-signal-intensity mass occupying more than three-quarters of the rectal wall (TIL, C4). rDWI image (b=800 s/mm^2^) **(B)**, ADC color map (ADC= 0.98×10^−3^ mm^2^) **(C)**, T2*-weighted image **(D)** and R2* color map (R2* = 40.13 Hz) **(E)** show ROI delineations of the corresponding tumor. The histopathological specimen **(F)** shows moderately differentiated rectal adenocarcinoma invading the whole layer of rectal wall, accompanied by one intestinal lymph node metastasis.

The comparison of R2* and ADC values and their degree of correlation with different histopathological factors are summarized in **Tables 2** and **3**. Higher R2* values significantly correlated with higher tumor T stage, lymph node involvement, lower histological differentiation, high CEA level, the presence of EMVI and tumor deposit (**Figures 3** and **4**). Significant positive correlations were found between R2* and higher histopathological factors (r = 0.374 ~ 0.673, all p<0.05). However, there were no significant differences between R2* values and other histopathological factors (normal/high CA19-9 level, the status of CRM and TIL) (r = 0.240, 0.256 and 0.265, p>0.05).

**Table 3:** Correlation of R2* and apparent diffusion coefficient with histopathological factors of rectal cancer.

Histopathological factor	R2* (Hz)		ADC (×10^−3^ mm^2^)	
	r-value	p-value	r-value	p-value
pT category	0.673	**<0.001**	−0.588	**<0.001**
pN category	0.385	**0.004**	−0.299	**0.028**
Tumor grade	0.460	**<0.001**	−0.519	**<0.001**
CEA level	0.388	**0.004**	−0.441	**0.001**
CA19-9 level	0.240	0.080	−0.211	0.126
CRM	0.256	0.061	−0.394	**0.002**
EMVI	0.479	**<0.001**	−0.574	**<0.001**
TIL	0.265	0.052	−0.396	**0.003**
Tumor deposit	0.374	**0.005**	−0.497	**<0.001**

CRM, circumferential margin; EMVI, extramural vascular invasion; TIL, range of tumor involvement in the circumference lumen; ADC, apparent diffusion coefficient.

The bolded numbers in the “p-value” columns indicated there were significant differences.

Meanwhile, lower ADC values inversely correlated with higher tumor T stage, lymph node involvement, lower histological differentiation, high CEA level, the positive status of CRM, the presence of EMVI and tumor deposit (r = −0.588 to −0.299, all p<0.05) (**Figures 3** and **4**), with the exception of high CA19-9 level (r = −0.211, p=0.126).

### Diagnostic Performances of R2* and ADC Values for Differentiation of Histopathological Prognostic Factors

**Figure 5:**
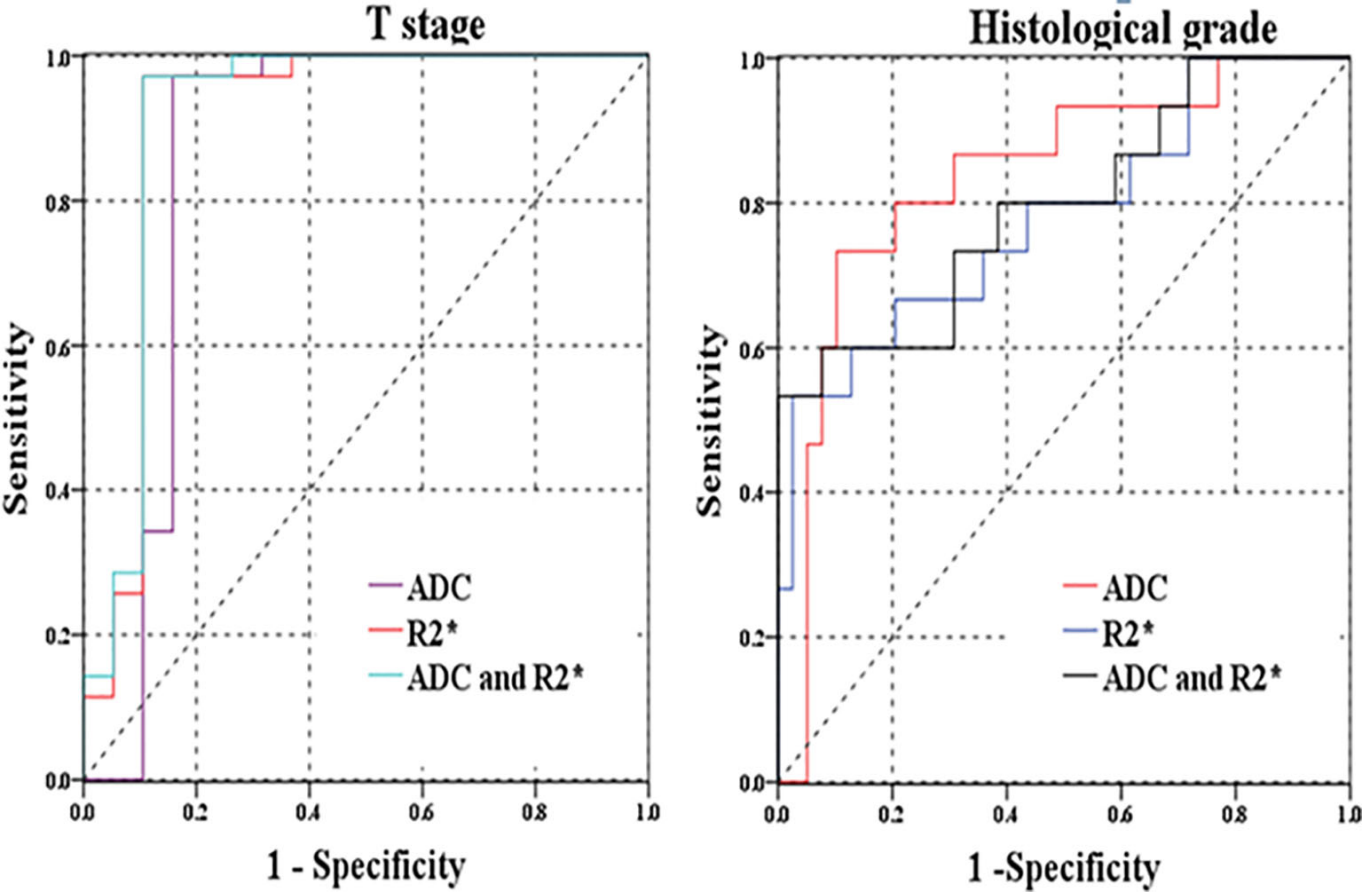
**(A)** ROC curves of R2* and ADC for distinguishing T stage (pT1-2 versus pT3-4); **(B)** ROC curves of R2* and ADC for discriminating histological grades (well/moderately-differentiated versus poor-differentiated rectal cancer).

In the ROC analysis, R2* and ADC exhibited different diagnostic efficacies for T category, N category, tumor grade, CEA level, the presence of EMVI and tumor deposit (**Table 4** and **Figure 5**). The AUC ranges for discrimination of above histopathological factors were 0.724–0.907 for R2* and 0.674–0.887 for ADC. However, no significant discriminative power was found for R2* in distinguishing normal/high CA19-9 level (AUC: 0.689, p = 0.080), negative/positive status of CRM (AUC: 0.720, p= 0.062) and TIL involvement (AUC: 0.679, p = 0.053), and ADC in differentiating normal/high CA19-9 level (AUC: 0.651, p = 0.125).

**Table 4:** Diagnostic performances of R2* and apparent diffusion coefficient for differentiation between different histopathological categories of rectal cancer.

Histopathological factor	Parameter	Cutoff	AUC	Sensitivity (%)	Specificity (%)	p-value
pT category	R2*	40.408	0.907 (0.793–1.000)	97.10	89.50	**<0.001**
	ADC	1.034	0.856 (0.708–1.000)	84.20	97.10	**<0.001**
	R2*+ADC	—	0.913 (0.803–1.000)	97.10	89.50	**<0.001**
pN category	R2*	44.239	0.724 (0.589–0.858)	50.00	76.70	**0.005**
	ADC	1.003	0.674 (0.529–0.818)	50.00	87.50	**0.030**
	R2*+ADC	—	0.714 (0.577–0.851)	62.50	76.70	**0.007**
Tumor grade	R2*	51.081	0.781 (0.633–0.929)	53.30	97.40	**0.001**
	ADC	0.899	0.834 (0.709–0.959)	89.70	73.30	**<0.001**
	R2*+ADC	—	0.797 (0.653–0.941)	53.30	100.00	**0.001**
CEA level	R2*	41.938	0.738 (0.606–0.870)	88.90	55.60	**0.005**
	ADC	0.960	0.770 (0.647–0.893)	55.60	100.00	**0.001**
	R2*+ADC	—	0.756 (0.629–0.883)	94.40	58.30	**0.002**
CA19-9 level	R2*	42.233	0.689 (0.521–0.858)	90.90	51.20	0.080
	ADC	0.954	0.651 (0.498–0.804)	46.50	90.90	0.125
	R2*+ADC	—	0.685 (0.525–0.845)	90.90	58.10	0.060
CRM	R2*	51.081	0.720 (0.510–0.931)	57.10	89.40	0.062
	ADC	0.923	0.839 (0.716–0.962)	59.60	100.00	**0.004**
	R2*+ADC	—	0.739 (0.539–0.938)	57.10	89.40	**0.043**
EMVI	R2*	42.233	0.824 (0.685–0.963)	84.60	51.20	**<0.001**
	ADC	0.899	0.887 (0.774–1.000)	90.20	84.60	**<0.001**
	R2*+ADC	—	0.816 (0.666–0.967)	69.20	92.70	**0.001**
TIL	R2*	51.081	0.679 (0.519–0.839)	19.50	92.30	0.053
	ADC	1.037	0.767 (0.608–0.926)	69.20	82.90	**0.004**
	R2*+ADC	—	0.767 (0.608–0.926)	85.40	69.20	**0.004**
Tumor deposit	R2*	51.081	0.760 (0.603–0.917)	50.00	92.90	**0.006**
	ADC	0.922	0.845 (0.728–0.962)	66.70	91.70	**<0.001**
	R2*+ADC	—	0.774 (0.620–0.927)	58.30	88.10	**0.004**

CRM, circumferential margin; EMVI, extramural vascular invasion; TIL, range of tumor involvement in the circumference lumen; ADC, apparent diffusion coefficient; AUC, area under the curve.

The units for R2*and ADC are Hz and 10^−3^ mm^2^ respectively.

The bolded numbers in the “p-value” columns indicated there were significant differences.

In comparison with individual R2* or ADC parameter, the combination of R2* and ADC parameters did not significantly improve the diagnostic performances for differentiation of related histopathological factors (p>0.05). The highest AUC of combined R2* and ADC parameters was 0.913(0.803–1.000) for discrimination of T category, with relatively higher specificity of 89.50% and highest sensitivity of 97.10% than those for other prognostic factors (**Figure 5**).

## Discussion

In our study, the T2*-weighted imaging and reduced field-of-view diffusion-weighted imaging of rectal cancer were successfully utilized to measure entire tumor volume with excellent interobserver agreement between readers. We found that both R2* and ADC parameters significantly correlated with some histopathological prognostic factors of rectal cancer, including T category, lymph node involvement, histological differentiation, CEA level, the presence of EMVI and tumor deposit, with additional association of ADC parameter with the status of CRM and TIL. Therefore, R2* and ADC metrics, derived from T2*-weighted imaging and rDWI respectively, could be promising imaging biomarkers for prediction of treatment-related prognosis of rectal cancer.

Our study demonstrated that significantly higher R2* values were found in patients with higher T stage, metastatic lymph node, higher histological grade, high CEA level, the presence of EMVI and tumor deposit. The R2* value exhibited significantly positive correlation with above prognostic factors. These prognostic factors indicated that rectal lesions were characterized by more invasive behaviors. Similar findings were reported by previous studies. Wang et al. (26) found that R2* value could be used to distinguish the histological grade and T stages of bladder cancer. Zhang et al. (22) concluded that histogram analysis of R2* could differentiate low- from high-grade clear cell renal cell carcinoma. These phenomena might be ascribed to rapid proliferation of tumor cells and growth of tumor tissues with more aggressive manner, resulting in tumors with increasing hypoxic status (27). The hypoxic status of rectal cancer would lead to an increase in the deoxyhemoglobin content and R2* values. Moreover, the tumor hypoxia of rectal cancer would further bring about angiogenesis, which is a fundamental process in tumor growth and invasion (28). So higher R2* values were often linked with more aggressiveness of rectal cancer, which was indicated by the results of our study.

However, R2* value did not correlate with CA19-9 level, the status of CRM or TIL involvement. As for CA19-9 level, possible reason could be that it might not reflect the actual status of rectal cancer with low sensitivity or specificity. As for the status of CRM and TIL involvement, more patients were needed to investigate the correlation between R2* value and these prognostic factors.

With respect to ADC measurement from rDWI, ADC values were found to have correlation with almost all the histopathological factors, except CA19-9 level. Lower ADC values were recorded in rectal cancer with more aggressive features. It was reported that elevated CEA level could be associated with tumor aggressiveness (29). The ADC values may reflect the aggressiveness of tumors with complex microstructures. The more aggressive rectal cancer was often associated with increased cellular density, abnormal gland formation and conspicuous variation of nuclear pleomorphism histopathologically, resulting in lower ADC values. Moreover, contradicting findings (30–32) about ADC measurement were noted in previous investigations regarding T stage, nodal involvement, histological differentiation, CEA level and lympho-vascular invasion (LVI). A recent large meta-analysis (33) about associations between ADC and numerous clinical and histochemical parameters in rectal cancer was published. It indicated that ADC can reflect expression of Ki-67 but no other relevant markers (tumor stages, grades and KRAS status). These inconsistent results might be imputed to the following factors: the disparities of scanning parameters among different DWI protocols, such as magnetic field strength, coil system, and b value; ADC measurement influenced by diverse ROI positioning protocols due to tumor heterogeneity (34). The ADC quantification in our study was based on rDWI technique, which could improve image quality, reduce artifacts and distortions for imaging of rectal cancer. This could explain the results obtained were different from previous studies of rectal cancer.

Our study had some limitations. First, our study type was retrospective with inevitable selection bias. Second, the relative small sample size and uneven distribution of patients with different histopathological features might influence the application of our results. Third, the inevitable multiple testing between R2*/ADC parameters in relation with different prognostic factors should be noted with adjustments by Bonferroni corrections. Finally, R2* values from T2*-weighted imaging are influenced by magnetic field strength, blood oxy/deoxyhemoglobin levels, blood volume and tumor vasculatures, which should also be considered for clinical practice of T2*-weighted imaging.

In conclusion, both R2* from T2*-weighted imaging and ADC from rDWI might act as potential imaging biomarkers for differentiation of histopathological prognostic factors of rectal cancer. The T2*-weighted imaging (R2*) could provide quantitative information for tumor characterization of rectal cancer.

## Data Availability Statement

The raw data supporting the conclusions of this article will be made available by the authors, without undue reservation.

## Ethics Statement

This retrospective study was conducted under the approval of the Ethics Committee of Tongji Hospital, Tongji Medical College, Huazhong University of Science and Technology. Written informed consent for participation was not required for this study in accordance with the national legislation and the institutional requirements. Written informed consent was not obtained from the individual(s) for the publication of any potentially identifiable images or data included in this article.

## Author Contributions

ZL conceived the experiment. DH designed the experiment. XH performed the experiment. YL analyzed the data. YS performed the statistical analysis. IK contributed to manuscript editing and reviewing. YP wrote the original draft. All authors contributed to the article and approved the submitted version.

## Funding

This work was supported by the National Natural Science Foundation of China (No. 81771801, 81701657, 81801695, 82071889, 82071890, 82001786).

## Conflict of Interest

The authors declare that the research was conducted in the absence of any commercial or financial relationships that could be construed as a potential conflict of interest.
